# Cost-effectiveness analysis of introducing HTLV-1 testing in South Africa

**DOI:** 10.1186/1742-4690-12-S1-P81

**Published:** 2015-08-28

**Authors:** Wendy Sykes, Charl Coleman, Genevieve Beck, Jabu Mhlanga, Carol Hlela, Brian Custer, Edward L Murphy, Marion Vermeulen

**Affiliations:** 1 South African National Blood Service, Johannesburg and Durban, South Africa; 2 University of Cape Town, Cape Town, South Africa; 3 Blood Systems Research Institute, San Francisco, California, USA; 4 University of California San Francisco, San Francisco, California, USA

## 

We have previously reported a 2013 cross-sectional study of HTLV prevalence among 46,765 South African blood donors. Confirmed HTLV-1 prevalence was 0.16% in Black donors, 0.02% in both White and Coloured donors and 0% in south Asian donors, for an overall prevalence of 0.062% extrapolated to the current blood donor population. Using these data we estimated the cost effectiveness of potential HTLV screening strategies in preventing transfusion transmitted HTLV-1 infection (TTI). Five blood donor screening strategies were considered: no screening; HTLV testing of every donation; HTLV testing each donor one time only; HTLV testing of new donors only; and universal filter leukodepletion without HTLV testing. The size of the population to be screened annually was 831,565 for universal screening, 507,054 for screening all donors once and 133,050 for screening new donors only. Using a prevalence of 0.062% and an assumed transmission efficiency of 10% because of buffy coat removal during red cell production, we calculated intervention and donor management costs and estimated the number of TTI prevented. There would be an estimated 58, 1, 1, 50 and 0.5 potential TTI cases under no screening, universal screening, screening all donors once, screening new donors only, and universal leukodepletion, respectively. The estimated costs per TTI prevented were $0, $31,574, $26,260, $60,391 and $5,151,680, respectively for the same strategies (at 11. SA Rand per 1 US dollar). Assuming the most cost-effective strategy of screening each donor once, it would cost the blood service approximately US$26,260 per TTI prevented in the first year, increasing to US$60,391 per TTI prevented in subsequent years as positives were culled from the donor population. Based upon these data, SANBS is carefully considering the costs and benefits of introducing HTLV screening in South Africa. This approach may be useful to other HTLV-endemic countries which do not currently test for HTLV.

